# Indigenous Yeasts from Rose Oil Distillation Wastewater and Their Capacity for Biotransformation of Phenolics

**DOI:** 10.3390/microorganisms11010201

**Published:** 2023-01-12

**Authors:** Mila Rusanova, Krasimir Rusanov, Veronika Butterweck, Ivan Atanassov

**Affiliations:** 1 AgroBioInstitute, Agricultural Academy, 1164 Sofia, Bulgaria; 2 Institute for Pharma Technology, School of Life Sciences, University of Applied Sciences and Arts Northwestern Switzerland, 4132 Muttenz, Switzerland

**Keywords:** rose oil distillation wastewater, indigenous *Cyberlindnera* yeast fermentation, phenolics biotransformation

## Abstract

The indigenous yeasts associated with the spontaneous fermentation of phenolic-rich rose oil distillation wastewater (RODW) generated after the industrial distillation of rose oil were studied. The ITS-rDNA sequence analysis of the samples collected from RODW fermented at semi-sterile conditions, a waste deposition lagoon and endophytic yeasts isolated from industrially cultivated *Rosa damascena* suggests that the spontaneous RODW fermentation is caused by yeasts from the genus *Cyberlindnera* found also as endophytes in the rose flowers. Phylogenetic analysis based on the nucleotide sequences of the translation elongation factor (TEF1α) and 18S- and 26S- rRNA genes further confirmed the taxonomic affiliation of the RODW yeast isolates with the genus *Cyberlindnera*. The RODW fermentation capacity of a selected set of indigenous yeast isolates was studied and compared with those of common yeast strains. The indigenous yeast isolates demonstrated a superior growth rate, resulting in a nearly double reduction in the phenolic content in the fermented RODW. The indigenous yeasts’ fermentation changed the RODW phenolics’ composition. The levels of some particular phenolic glycosides decreased through the depletion and fermentation of their sugar moiety. Hence, the relative abundance of the corresponding aglycons and other phenolic compounds increased. The capacity for the biotransformation of RODW phenolics by indigenous yeasts is discussed.

## 1. Introduction

Rose oil distilled from the flowers of *Rosa damascena* Mill. is one of the most valuable essential oils used as a base component in a wide range of high-quality perfumery and cosmetic products [1]. One kilogram of rose oil is produced by the water-steam distillation of about 3000 kg of freshly collected rose flowers. Prior to distillation, rose flowers are mixed with water in a 1:4 ratio (*w*/*v*), i.e., the distillation of 1 kg of rose oil requires a total of 12,000 L of water. The industrial water-steam distillation lasts about two and a half hours, and the boiled aqueous mixture of rose flower remedies remaining in the distiller tank is quickly discharged at the end of the distillation cycle. Taking into account the volume of the obtained distillate, the production of 1 kg rose oil results in the release of about 7500 L of liquid waste containing pieces of rose flower remedies. Over 80% of the discharged waste represents phenolic-rich rose oil distillation wastewater (RODW). Due to the high content of phenolics and their slow decomposition by microorganisms, phenolic-rich wastewaters, including RODW, are considered biopollutants. Thus, it is required to minimize their discharge into drainage systems and rivers [2,3].

Our previous research showed that RODW phenolics consist mainly of flavonoids including quercetin and kaempferol glycosides, ellagic acid and phenylethyl alcohol glycosides [2]. Analysis of the phenolic-enriched resin extract obtained from the RODW showed that it has high antioxidant activity [2,4], diverse biological activities and potential applications including skin whitening, due to the tyrosinase inhibition in skin cells [5], as well as the inhibition of cell proliferation for the potential treatment of psoriasis [6]. During the scale-up phase for the extraction of RODW phenolics, we observed that the hot RODW discharged from the rose oil distillers into the cooling pool started to ferment within 2–4 days, and was subject to spontaneous fermentation even when nearly boiling RODW samples were collected in sterile bottles following the RODW discharge. To our knowledge, the spontaneous fermentation of RODW discharged from rose oil distillation facilities has not been studied or reported so far. This raises the question of the cause of the fermentation and its impact on the composition of RODW phenolics. So far, bacterial, yeast and fungal fermentation have been successfully applied to the biotransformation and bioremediation of phenolic-rich wastewater from various sources and with various compositions. A notable example is the olive mill wastewater, which has been a subject of directional phenolic modifications and/or degradation via various microbial fermentations and treatments [7,8,9,10,11]. Indigenous yeasts and molds inhabiting the production sites relating to the processing of phenolic-rich plant products and their associated wastes offer a valuable source for the selection of isolates with a high fermentation capacity for phenolic-rich plant products and wastewater [12,13,14,15,16].

In this study, we investigated the yeast and fungal biodiversity relating to the early fermentation following the hot RODW discharge from the distillers and isolated indigenous yeasts causing the spontaneous fermentation. The taxonomic affiliation of the isolated indigenous yeasts and their impact on the fermentation and the RODW’s phenolic composition were studied. The RODW fermentation rate and phenolic biotransformation caused by indigenous yeast isolates and common yeast strains were compared.

## 2. Materials and Methods

### 2.1. Sampling of Rose Oil Distillation Wastes

Two types of rose oil distillation liquid wastes were collected from a private distillery in the Rose Valley, Bulgaria, during the 2018 rose oil distillation campaign. The two types of wastes were designated as RODW (Rose Oil Distillation Wastewater) and DLRFR (Deposited Liquid Rose Flower Remedies). They correspond to the two types of waste processing practices employed in the industry (more detailed explanations of the waste processing practices are presented in the Discussion part). In short, the RODW sample is rose oil distillation wastewater made when the distillery uses a cooling pool and separates flower remedies, while the DLRFR sample is rose oil distillation wastewater made when the entire waste from the distillers is transferred directly to a lagoon located near the distillery. RODW samples were collected in a sterile glass bottle directly from the cooling pool immediately after the discharge of the hot liquid wastes from the rose oil distillers. Upon sample collection, a 500-mL glass bottle containing 400 mL of RODW was closed with a sterile cap and transferred to the lab. The cap was later replaced by a fine sterile nylon mesh under laminar flow, and the RODW was incubated at room temperature in sterile conditions for four days without agitation in order to allow the growth of indigenous microorganisms. Additionally, RODW from the cooling pool was collected in 1000 mL glass bottles and autoclaved at 121 °C for 20 min, resulting in sterile liquid RODW medium used for further cultivation experiments. DLRFR samples were collected in 50-mL sterile plastic containers from the waste lagoon located near the distillery.

### 2.2. Collection of Indigenous Yeast and Mold Isolates from RODW and DLRFR Samples

One milliliter portions of DLRFR and RODW samples were used for preparation of serial dilutions with sterile water. 100 microliters of the diluted samples were plated on solid RODW medium consisting of RODW and 20 g/L agar, with or without addition of 30 mg/L chloramphenicol for selective cultivation of yeasts and molds. The RODW solid medium was autoclaved at 121 °C for 20 min, without pH correction prior to sterilization. The cultures were incubated at 28 °C for 2–4 days in a BD 56 incubator (BINDER GmbH, Germany) until single colonies were obtained. Single colonies were transferred and repeatedly sub-cultured on fresh RODW, YPD (10 g yeast extract, 20 g peptone, 20 g dextrose, 20 g agar, *w*/*v*, pH 6.5) and PDA (39 g Difco^TM^ Potato Dextrose Agar, BD, France, *w*/*v*) media for further studies.

### 2.3. Isolation of Yeast and Mold Rose Flower Endophytes from Flower Buds of R. damascena Mill

**Figure 1:**
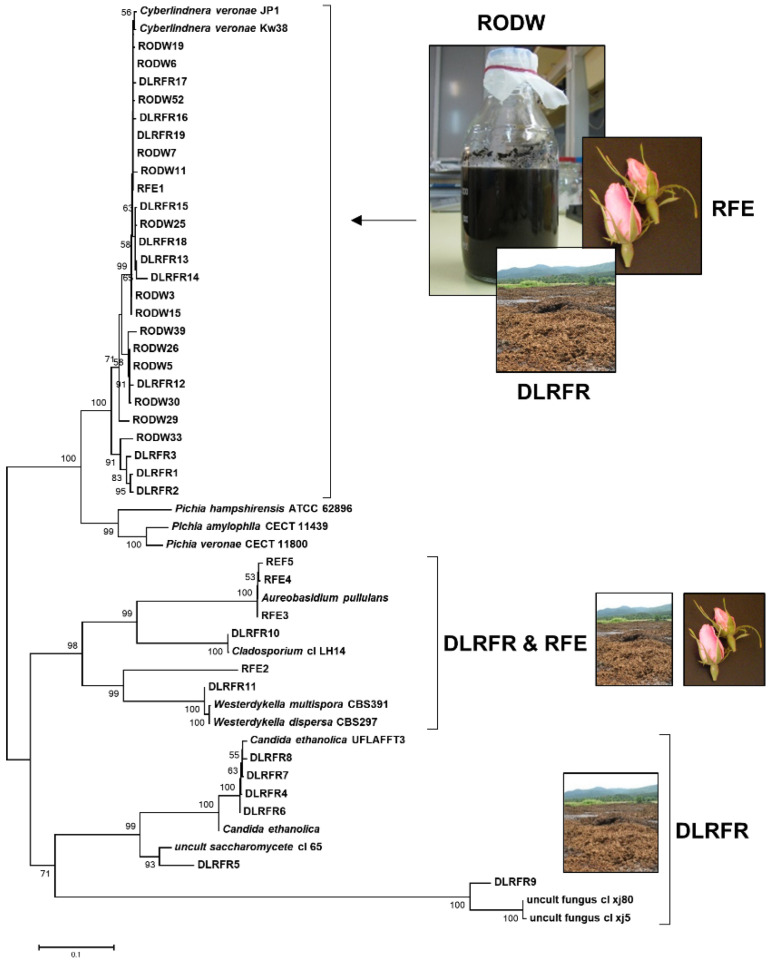
Molecular characterization of yeast and mold diversity. A neighbor-joining phylogenetic tree constructed from ITS sequences of RODW, DLRFR and RFE isolates and BLAST search identified sequences showing high level of homology. The percentage of replicate trees in which the associated taxa clustered together in the bootstrap test are shown next to the branches.

RFE (Rose Flower Endophytes) yeast and mold were isolated from unopened flower buds of *R. damascena* Mill (as shown in Figure 1) collected at the beginning of the active rose flowering from 11 different plantations in the Rose Valley of Bulgaria, at locations described by Rusanov et al. [17]. After sterilization with 70% ethanol for 30 s and 0.1% HgCl_2_ for 7 min, the flower buds were cut and the petals from the internal part of the flower bud were cultivated in Petri dishes with sterile YPD and PDA media. The cultures were incubated at 25 °C in a BD 56 incubator (BINDER GmbH, Germany) and observed for growth of microorganisms. Developing microorganisms were promptly transferred and sub-cultured on fresh nutrient media for further studies.

### 2.4. Common Yeast Strains Used for Fermentation of RODW

In parallel to the indigenous yeast and mold isolates, a set of 24 yeast strains kindly provided by the National Bank for Industrial Microorganisms and Cell Cultures (NBIMCC) at the University of Chemical Technology and Metallurgy (UCTM)—Sofia, Bulgaria, was used to study the RODW fermentation. The strains were *Candida zeylanoides* ATCC 24745, *Cryptococcus flavus* ATCC 10656, *Cryptococcus laurentii* ATCC 10668, *Debaryomyces hansenii* var. *hansenii* ATCC 36239, *Geotrichum fermentans* ATCC 10675, *Pichia angusta* ATCC 34438, *Pichia anomala* ATCC 16763, *Pichia bispora* ATCC 10627, *Pichia carsonii* ATCC 24214, *Pichia guilliermondi* ATCC 9058, *Pichia jadinii* ATCC 9256, *Pichia trehalophila* ATCC 22307, *Pleurotus eryngii* CCRC 36213, *Rhodosporidium toruloides* ATCC 15385, *Rhodotorula lactosa* ATCC 18176, *Saccharomyces bayanus* DSM 70547, *Saccharomyces pastorianus* ATCC 12752, *Trichosporon montevideense* ATCC 90035, *Trichosporon cutaneum* ATCC 28592, *Yarrowia lipolytica* ATCC 18942, *Zygoascus hellenicus* ATCC 15542, *Zygosaccharomyces rouxii* ATCC 14679, *Zygosaccharomyces rouxii* DSMZ 7525 and *Zygosaccharomyces bisporus* ATCC 52405. Each of the yeast strains was cultivated on YPD and RODW solid media and further used in the studies.

### 2.5. Molecular Identification of Yeast and Mold Isolates from RODW, DLRFR and RFE Samples

The yeast and mold isolates were taxonomically identified using PCR amplification and direct sequencing of the ITS region of nuclear rRNA genes. The primers used for PCR amplification of the ITS region were ITS1 5′-TCCGTAGGTGAACCTGCGG and ITS4 5′-TCCTCCGCTTATTGATATGC [18]. The PCR amplification was performed using the following program: 3 min at 95 °C, followed by 33 cycles of 95 °C for 15 s, 53 °C for 30 s, 72 °C for 1 min and final extension at 72 °C for 3 min on a QB-96 thermal cycler (Quanta Biotech Ltd., Surrey, UK). The obtained PCR products were separated in 1.2% agarose gel, purified from the gel using GeneJET Gel Extraction and DNA Cleanup Micro Kit (Thermo Scientific, Waltham, MA, USA) and commercially sequenced (Macrogen, Inc., Seoul, Republic of Korea). The obtained ITS sequences were assembled using Vector NTI 10 (Life Technologies, Carlsbad, CA, USA) and used for BLAST search against sequences deposited in GenBank to determine their close relatives and approximate taxonomic affiliations. The obtained ITS sequences, together with selected pools of GenBank retrieved sequences, were subjected to phylogenetic analyses using MEGA 4 [19].

The taxonomic affiliation of selected yeast RODW isolates was further studied based on a phylogenetic analysis of the sequences of their 18S-rRNA, 26S-rRNA and TEF1α gene regions. Two primer pairs were used for amplification of the 26S-rRNA region (Pair 1: NL-1F 5′-GCATATCAATAAGCGGAGGAAAAG and NL-E27R 5′-TGACGAGGCATTTGGCTACC; Pair 2: NL-1611 F 5′-CCGCAGCAGGTCTCCAA and ETS-2AR 5′-GATCGTAACAACAAGGCTACTCTA, [20]), with one primer pair for amplification of the TEF1α region (983F 5′-GCYCCYGGHCAYCGTGAYTTYAT and 2218R 5′-ATGACACCR ACRGCRACRGTYTG, [21]) and one primer pair for amplification of the 18S-rRNA region (NS1 F 5′-GTA GTC ATA TGC TTG TCTC and NS8 R 5′-TCC GCA GGT TCA CCT ACG GA, [18]). The PCR amplifications were performed using the following program: 3 min at 95 °C, followed by 33 cycles of 95 °C for 15 s, 57 °C for 30 s, 72 °C for 1 min 45 s and final extension at 72 °C for 3 min on a QB-96 PCR thermal cycler (Quanta Biotech Ltd., UK). The obtained PCR products were separated in 1% agarose gel, purified from the gel and cloned in the pJET 1.2 plasmid vector using a CloneJet PCR Cloning kit (Thermo Scientific). The cloned 18S-, 26S- and TEF1α fragments were commercially sequenced (Macrogen, Inc). The obtained 18S-, 26S- rRNA and TEF1α sequences were assembled using Vector NTI 10 (Life Technologies) and used for BLAST search against sequences deposited in GenBank. The obtained sequences, together with selected pools of GenBank retrieved sequences, were subjected to phylogenetic analyses using MEGA 4 [19].

### 2.6. Fermentation of RODW with Indigenous Yeast Isolates and Common Yeast Strains

A single colony from each of the tested indigenous yeast RODW isolates and common yeast strains was used to initiate overnight cultures by inoculating 5 mL YPD medium and incubating at 28 °C and 190 rpm in a Climo-Shaker (Kuhner AG, Birsfelden, Switzerland). One milliliter of each overnight culture diluted to optical density OD_580_ = 1 was used for inoculation of 100 mL sterile liquid RODW media and incubated at 28 °C and 190 rpm in a Climo-Shaker (Kuhner AG, Birsfelden, Switzerland). Cultivation was performed in parallel with a control of sterile liquid RODW media. The growth rate and content of total phenols and reducing sugars in the cultures were studied through analyses of samples taken every two hours between 14 and 22, 38 and 46, 62 and 70, 86 and 94, and 110 and 136 h of the cultivation. The growth rate was monitored through measurement of the optical density (OD_580_) of the samples on a Cintra 20 UV-Vis spectrophotometer (GBC-Scientific Equipment Ltd., Braeside, Australia). At the final time point, samples were also taken for HPLC-UV analysis of phenolic composition. The comparative cultivation of RODW isolates and common yeast strains was performed in the same way using parallel cultures. The OD_580_ growth rate was monitored in the samples collected at time points 0, 16, 18, 20, 22, 24, 40, 42, 44, 48 and 64 h of the cultivation. All experiments were performed in triplicate. Graph plots were generated using Microsoft Excel 2013.

### 2.7. Phenolics Extraction from RODW Samples

Phenolics were extracted from RODW samples using 10 mL PD-10 columns (GE Healthcare, USA) filled with 1 g of Sepabeads^TM^ SP207 resin (Mitsubishi Chemical, Tokyo, Japan) purchased from Sigma-Aldrich. Prior to extraction, the resin was activated by addition of 4 mL of methanol for one hour, washed with ultrapure water, treated with 8 mL of 1M NaOH and washed three times with 10 mL of ultrapure water. Ten milliliters of either fermented or unfermented RODW samples were filtered with a 100-μm nylon mesh, loaded to the column and mixed using a Laboshake shaker (C. Gerhardt GmbH & Co. KG, Königswinter, Germany) at 180 rpm for 30 min. The phenolics adsorbed on the resin were collected after draining the column washed three times with 10 mL ultrapure water and eluted two consecutive times with 2 mL of 95% ethanol. The two eluates were combined, giving a RODW phenolic extract sample for further analysis.

### 2.8. Analytical Methods

#### 2.8.1. Total Phenolic Content

The total phenolic content was determined using the Folin-Ciocalteu method [22], adapted for a microplate reader. Briefly, 10 μL of the sample or standard (gallic acid) were mixed with 790 μL of distilled water and 50 μL of Folin-Ciocalteu’s phenolic reagent (Sigma-Aldrich, St. Louis, MO, USA) in 96 Nunc^®^ DeepWell™ plate (Sigma-Aldrich) and, after vortexing, were incubated for 5 min at room temperature. Then, 150 microliters of 20% Na_2_CO_3_ were added, and the samples were vortexed and incubated for 2 h at room temperature. Absorbance was measured at 670 nm using an LKB 6060–006 plate reader (LKB Vertriebs GmbH, Vienna, Austria). The results are expressed as gallic acid equivalents (GAE, mg/L).

#### 2.8.2. Reducing Sugar Content

The amount of reducing sugars was determined using the DNS method [23] adapted for a microplate reader. Briefly, 100 μL of the sample or standard (D-glucose) were mixed with 100 μL of DNS reagent (7.06 g of dinitrosalicylic acid, 13 g of NaOH, 200 g of Na-K tartrate, 5 mL of phenol, 5.53 g of sodium sulfite and distilled H_2_O to a final volume of 1 L) in a 96 Nunc^®^ DeepWell^TM^ plate (Sigma-Aldrich). After vortexing, the samples were heated at 94 °C for 15 min in a WNB 45 water bath (Memmert GmbH + Co. KG, Schwabach, Germany) and then quickly placed on ice. One milliliter of ultra-pure water was added to the cooled samples. Absorbance was measured at 540 nm using an LKB 6060–006 plate reader (LKB Vertriebs GmbH, Vienna, Austria). The amount of reducing sugars is expressed as D-glucose equivalents (mg/L).

#### 2.8.3. HPLC/UV Analysis

Two milliliters of each RODW extract sample were placed in a 15-mL Falcon tube and evaporated at 40 °C for 6 h using a CentriVap Benchtop Vacuum Concentrator (Labconco, Kansas City, MO, USA) for ethanol removal. The remaining water extract was frozen in liquid nitrogen and lyophilized using an Alpha 1-2 LD Plus freeze dryer (Martin Christ, Osterode am Harz, Germany). The dry RODW extracts were dissolved in 10% aqueous DMSO containing 3 mg/mL TBHQ (Sigma-Aldrich) as an internal standard to a final concentration of 1 mg/mL phenolics and filtered through a 0.45-μm MS^®^ nylon filter (Membrane Solutions). The HPLC analysis of the extracts was carried out on a Hitachi Elite LaChrom HPLC system consisting of an L-2130 pump and L-2420 UV-Vis detector operated by EZ Chrom Elite ver. 3.3.2 SP2 (Agilent Technologies, Santa Clara, CA, USA) and using H_2_O (A) and acetonitrile (B) as mobile phases, both containing 1% acetic acid. Twenty microliters of each sample were manually injected and analyzed on an Inertsil^®^ ODS-4 (4.6 × 250 mm, 5 μm, GL Sciences) column at room temperature using the following gradient program: 0 min 10% B, 0–40 min to 46% B, 40–42 min to 100% B, 42–52 min 100% B isocratic, 52–54 min to 10% B. UV absorbance was recorded at 254 nm. The phenolic compounds were identified by comparing their relative retention times with those of authentic compounds, purified and LC-MS analyzed in a previous study by Rusanov et al. [2]. All chromatograms were integrated using the EZ Chrom Elite ver. 3.3.2 SP2 (Agilent Technologies) software. The peak areas of the analyzed compounds were normalized to the area of the internal standard. The changes in the content of individual compounds following RODW fermentation were evaluated through calculation of the relative change of the content of each compound, expressed as percentage of the amount in the control sample (RODW extract without yeast inoculation).

#### 2.8.4. Statistical Analysis

Hierarchical clustering of yeast strains and compounds based on data for the relative changes of compounds in the extract following fermentation of RODW compared to a control of non-fermented RODW was performed with the help of SPSS Statistics ver. 25 (IBM, Armonk, NY, USA) using Euclidean distances as a measure and the nearest neighbor algorithm for building the dendrograms. Analysis of variance (ANOVA) including Tukey post-hoc test was performed using SPSS Statistics ver. 25 (IBM, USA).

## 3. Results

### 3.1. Molecular Characterization of Yeast and Mold Diversity of Rose Oil Distillation Wastes and Rose Flower Endophytes

A comparative molecular characterization of the yeast and mold diversity relating to the two types of liquid waste samples from industrial rose oil distillation, as well as the flower endophytes isolated from industrially cultivated *R. damascena* plants, was carried out using ITS sequence-based taxonomic identification. The liquid waste samples included a RODW sample collected immediately after the discharge of the hot liquid wastes from the rose oil distillers in the cooling pool and a DLRFR sample collected from the liquid part of the rose oil distillation wastes deposited in a waste lagoon. Two sets of 96 yeast and mold colonies derived from each RODW and DLRFR sample were selected for the study, following the cultivation of serial dilutions of the samples on RODW solid media. The ITS sequence analysis of the yeast and mold isolates derived from these colonies resulted in the identification of a total of 14 RODW and 19 DLRFR ITS sequences. In a parallel experiment, the in vitro cultivation of petal tissue from 88 unopened flower buds from *R. damascena* plants resulted in the identification and collection of only five endophytic yeast and mold isolates. Their ITS sequences were also included in the study, designated as RFE. The obtained ITS sequences were further used for a BLAST search against sequences deposited in GenBank, and the ITS sequences with a high level of homology and known taxonomic affiliations were retrieved. All of the ITS sequences were the subject of phylogenetic analysis. The constructed phylogenetic tree shows three main clusters of ITS sequences (Figure 1). The largest “RODW& DLRFR& RFE” cluster involved all the sequences of the RODW group and part of the sequences from the other two groups. The ITS sequences included in this cluster were closely related to the *Cyberlindnera* yeast species. The other two clusters were less populated and involved ITS sequences from the DLRFR and RFE groups, as well as the DLRFR group alone.

**Figure 2:**
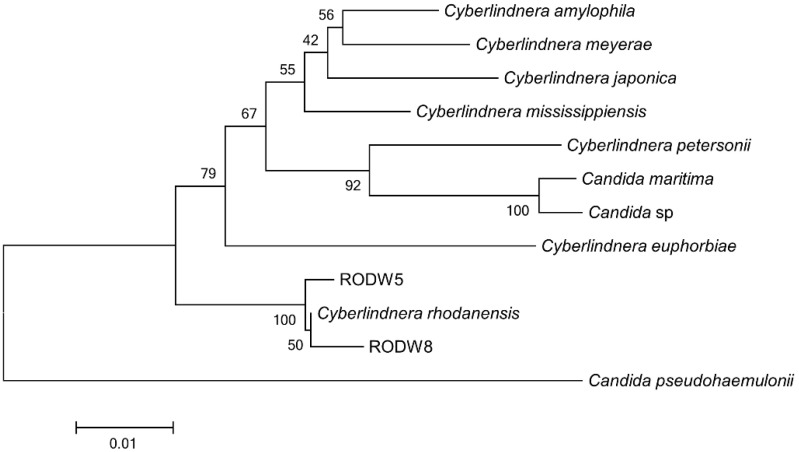
Taxonomic affiliation of indigenous yeast RODW isolates. A neighbor-joining phylogenetic tree constructed from 18S-rRNA, 26S-rRNA and TEF1α sequences of RODW5 and RODW8 isolates and BLAST search-identified sequences showing a higher level of homology.

The taxonomic affiliation of RODW isolates with the yeast genus *Cyberlindnera* was further confirmed through multigene sequence analysis involving sequences of the 18S-rRNA, 26S-rRNA and translation elongation factor 1α (TEF1α) genes. The phylogenetic tree was constructed from the sequences of two indigenous yeast isolates, RODW5 and RODW8, and the sequences identified by the BLAST search showing a high level of homology. The phylogenetic tree demonstrated a close affiliation of the multigene sequences of the RODW isolates with *Cyberlindnera rhodanensis*, as well as with a larger cluster involving other *Cyberlindnera* species (Figure 2).

### 3.2. Cultivation of Indigenous Yeast RODW Isolates in RODW Media

**Figure 3:**
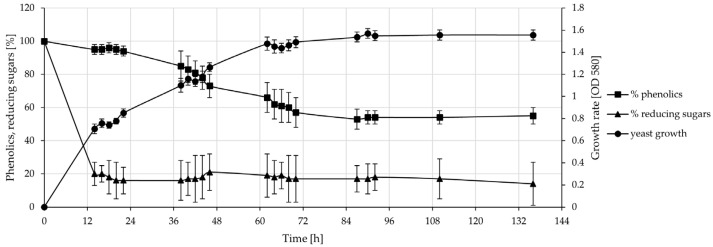
Fermentation of RODW by indigenous yeast RODW isolates. Mean data for 23 isolates for a period of 136 h. Left ordinate represents the phenolics and reducing sugar content as a percentage of their initial content in RODW prior to fermentation. Right ordinate presents the yeast growth rate through optical density (OD_580_) of the cultures. The RODW fermentations by the tested indigenous RODW isolates were performed in triplicate. Each point is an average ± SD of results from RODW fermentation by 23 different indigenous yeast isolates.

Twenty-three indigenous RODW yeast isolates, including RODW5 and RODW8, were cultivated in sterile RODW as a culture medium in shake-flask cultures. The dynamics of fermentation were studied through the periodic measurement of the growth rate, reducing sugars and the phenolic content for each culture for a period of 136 h post-inoculation, presented in Figure 3. The parallel cultivation of the yeast RODW isolates showed no significant differences in the growth rate, sugar fermentation and changes in the total phenolic content of the cultures. The active growth of yeast isolates in the RODW medium lasted for about 62–70 h. After that, the cultures entered the stationary phase. About 80% of the reducing sugars available in the RODW were fermented by the yeasts within the first 14 h of cultivation, with no significant changes in their content after that. On the contrary, the biotransformation and fermentation of the phenolics was slower and took place within 68–70 h after the inoculation. Additionally, the fermentation resulted in a smaller reduction in the phenolic content compared to the sugars. The phenolics decreased to about 60% of their initial content at the stationary phase.

### 3.3. Cultivation of Indigenous RODW Isolates and Common Yeast Strains in RODW Media

**Figure 4:**
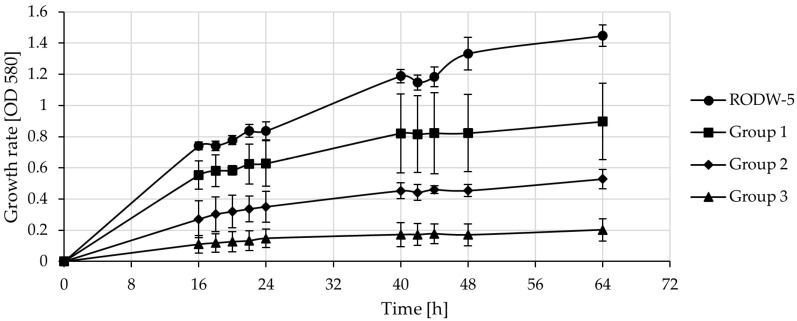
Comparative cultivation of common yeast strains and indigenous RODW-5 isolate in RODW media. The growth curves represent the three groups of common yeast strains formed after clustering analysis based on the growth rate of each strain. All RODW fermentations were performed in triplicate. Each “RODW-5” point is an average ± SDs of the results from three independent RODW-5 cultivations. Each “Group 1,” “Group 2” and “Group 3” point is an average of the results of the RODW fermentations by the tested common yeast strains from the group, as follows. Group 1 strains: *Pichia angusta*, *P. bispora*, *P. guillermondi*, *P. jadinii* and *Debaryomyces hansenii*; Group 2 strains: *Geotrichum fermentans*, *P. anomala*, *P. trehalophila*, *Zygoascus hellenicus* and *Zygosaccharomyces bisporus*; Group 3 strains: *Rhodosporidium toruloides*, *Saccharomyces byanus*, *S. pastorianus*, *Yarrowia lipolytica*, *Z. rouxii* and *Cryptococcus laurentii*.

Twenty-four common yeast strains derived from the culture collection of NBIMCC were tested in parallel cultivations for their capacity for RODW fermentation and compared to the indigenous yeast isolate RODW-5 (Figure 4). Seven of the tested strains did not grow in RODW for the studied period of 64 h. The remaining 17 strains were clustered in three groups according to the level and dynamics of their growth rate. The strains from Group 1 representing *Pichia angusta*, *P. bispora*, *P. guillermondi*, *P. jadinii* and *Debaryomyces hansenii* showed the highest rate of growth among the tested common yeast isolates. Group 2, represented by the five strains of *Geotrichum fermentans*, *P. anomala*, *P. trehalophila*, *Zygoascus hellenicus* and *Zygosaccharomyces bisporus,* showed a moderate level of growth, while Group 3, including the seven strains of *Rhodosporidium toruloides*, *Saccharomyces byanus*, *S. pastorianus*, *Yarrowia lipolytica*, *Z. rouxii* ATCC 14679, *Z. rouxii* DSMZ 7525 and *Cryptococcus laurentii,* grew at the slowest rate in RODW. However, none of the tested strains matched the growth rate of the indigenous isolate RODW-5 (Figure 4).

### 3.4. RODW Phenolic Biotransformation after Fermentation by Indigenous Rodw Yeast Isolates and Common Yeast Strains

The biotransformation of RODW phenolics after fermentation with the studied indigenous yeast isolates was evaluated through the extraction of phenolics from the culture media obtained after the parallel RODW fermentations with individual RODW yeast isolates described in part 3.2 above. Following the extraction of the phenolics through adsorption on microporous resin, the composition of the obtained phenolic extracts from the RODW cultures were HPLC-UV analyzed and compared with the phenolic composition of the extract from unfermented RODW. The obtained data showed no significant differences in the phenolic composition among the extracts obtained after the fermentation of RODW with the tested indigenous RODW yeast isolates. On the other hand, the fermentation with RODW yeast isolates caused significant changes in the abundance of part of the phenolic compounds, as presented in Table 1 (column RODW-5) and Supplementary Figure S1a. The obtained data for the relative changes in the phenolic compounds in the extract following fermentation by RODW-5 were subjected to further clustering analysis. The constructed dendrogram shown in Supplementary Figure S2 grouped the analyzed compounds into three main clusters with similar patterns of changes following RODW fermentation by the indigenous RODW-5 isolate. The first cluster included five compounds that decreased in abundance following fermentation. Three of them, isoquercitrin, kaempferol-3-O-rhamnoside and multiflorin A, were completely assimilated, whereas the abundance of the other two compounds, ellagic acid and astragalin, decreased about 6 times and 3.4 times, respectively. On the contrary, the concentrations of kaempferol and kaempferol-3-O-arabinoside from the second cluster increased, respectively, with a 4.7-fold and 5.45-fold relative increase in their content in the fermented RODW. The third cluster was the most populated, containing compounds with a moderate increase (about 2-fold) in their content, Table 1. Generally, the RODW fermentation by indigenous yeast isolates resulted in the elevated abundance of two aglycons, quercetin (1.7-fold) and kaempferol (4.7-fold), due to the assimilation of the sugar residue of some of their respective glycosides during the yeast fermentation.

In the second part of the study, the RODW phenolic biotransformation capacity of common yeast strains was evaluated through the HPLC-UV analysis of extracts from RODW culture media after fermentation with three yeast strains (*P. angusta*, *P. bispora* and *P. guillermondi*,) from Group 1 and three yeast strains (*G. fermentans*, *P. anomala* and *Z. hellenicus*) from Group 2, as described in part 3.3. The composition of the phenolic extracts from the RODW fermented by all six strains is presented in Table 1, and an HPLC chromatogram of the *G. fermentans* extract, are shown in Supplementary Figure S1b. There were significant differences in the composition of the obtained phenolic extracts involving changes in the abundance of particular phenolic compounds without the formation of new ones after the RODW fermentations of all the tested strains, as shown in Table 1 and Supplementary Figure S1b. The changes in the RODW phenolic composition after the fermentation by *Pichia bispora* included a higher number of compounds and an extended range of changes in the compound abundance in comparison to the other common yeast strains. This was confirmed by the dendrogram constructed via hierarchical clustering of the yeast strains based on the relative changes in the abundance of phenolics, as shown in Supplementary Figure S3. The dendrogram demonstrates the closer clustering of five of the tested common yeast strains, the distant location of the *Pichia bispora* strain and an intermediate position of the RODW-5 isolate. Similarly to the fermentation by the RODW-5 isolate, the abundance of the aglycons kaempferol and quercetin increased from 1.55- to 11.82-fold and from 1.4- to 6.3-fold, respectively, for all the tested strains. However, no particular compounds were completely assimilated, in contrast to the fermentation with the RODW-5 isolate. This suggests that the studied indigenous *Cyberlindnera* yeast isolate is better adapted to the fermentation of phenolic-rich RODW wastewater. Similarly to the RODW fermentation by indigenous yeast isolates, the RODW fermentation by common yeast strains resulted in changes in the relative abundance of part of the phenolic compounds, but not in the biosynthesis of new compounds.

## 4. Discussion

The industrial water-steam distillation of rose oil generates large volumes of waste and a boiled aqueous mixture of rose flower remedies. Currently, the industry employs two main practices for processing the generated waste. One of these practices involves the discharge of the entire boiled aqueous waste mixture from the distillers into a cooling pool, combined with the separation and removal of the flower remedies and subsequent release of the cooled phenolic-rich wastewater into the drainage system and nearby rivers. The whole process takes less than 24 h. Since the distillers are discharged once or several times per 24 h during the rose harvest campaign, the temperature of the aqueous waste in the cooling pool change with repeated cycles, from around 70 °C to 90 °C to nearly ambient temperature, before the following release. The second practice involves the direct transfer and deposition of the entire boiled aqueous waste mixture into a lagoon located next to the distillation facility. Similarly to the above, the local temperature at the spot where the hot waste is poured out into the lagoon changes with repeated cycles, whereas the temperature of the rest of the lagoon remains ambient. The aqueous part of the waste is rich in flower-derived sugars and phenolics [2]. Our preliminary observations showed that this wastewater undergoes spontaneous yeast fermentation, starting soon after its release from the distiller and later resulting in a change of the wastewater color to black.

In this study, we carried out the ITS-based taxonomic identification of yeast and mold isolates relating to the spontaneous fermentation of the wastewater derived from both waste-processing practices. Therefore, the study involves the analysis of a RODW sample collected in a sterile bottle from the hot wastewater of the cooling pool, and a DLRFR sample representing the aqueous part of the waste collected at the place of discharge of the waste in a lagoon. The results from the ITS analysis indicated that the early spontaneous RODW fermentation is dominated by a community of indigenous yeasts of the genus *Cyberlindnera*. The phylogenetic tree of ITS sequences showed that a large part of DLRFR isolates were also yeasts representing *Cyberlindnera* sp. clustering together with RODW isolates. The rest of the DLRFR isolates were affiliated with other yeast and mold genera and most likely represent the members of yeast and mold communities of the lagoon associated with the later stages of the long-term fermentation of the deposited rose oil distillation waste.

The obtained results suggest a scenario where the early spontaneous fermentation of the wastewater is caused by a community of indigenous yeasts of *Cyberlindnera* sp. which possess a high capacity for RODW fermentation. These indigenous yeasts permanently inhabit the environment of the distillation facilities and are well-adapted to the phenolic-rich wastewater, resulting in the high rate of its spontaneous fermentation. This was supported by the results from the RODW fermentation experiments, where the RODW-5 isolate showed a superior fermentation capacity in comparison to all the other tested common yeast strains. Additionally, the finding that *R. damascena* flowers harbor *Cyberlindnera* sp. endophyte yeasts that clustered together with the RODW isolates provides ground to speculate that the indigenous *Cyberlindnera* yeasts which inhabit the distillation facilities could originate from rose flower endophytes brought with the rose flowers and adapted to the discharged phenolic-rich wastewater. Similar scenarios of the spontaneous fermentation of plant phenolic-rich products and aqueous mixtures by indigenous yeasts were reported for other plant processing industries as well, such as the spontaneous grape must fermentation by indigenous yeasts inhabiting the winery and vineyards [12,13,24], and the spontaneous fermentation of cocoa pulp [14], olive mill wastewater [7,10,11], coffee processing wastes [25], tea [26] and others [16].

The multisequence phylogenetic analysis confirmed the taxonomic affiliation of the RODW isolates with the *Cyberlindnera* genus and their closer affiliation with the *Cyberlindnera rhodanensis* species. The genus *Cyberlindnera* was introduced as a replacement name for the genus *Lindnera* and currently accommodates over thirty species, some of which are associated with the fermentation of plant biomass and wastes, as well as phenolic-rich beverages and food products [26,27,28,29,30,31,32,33,34]. In the present study, the fermentation of RODW by the tested indigenous yeast isolates resulted in the fermentation of around 80% of the reducing sugars present in the RODW and a decrease in the phenolic content to around 60% of the initial concentration in the RODW.

Further analysis of the phenolic composition of the fermented RODW showed a diverse mode of changes in the abundances of different compounds, varying from the complete assimilation of isoquercitrin, kaempferol-3-O-rhamnoside and multiflorin A through the reduction of the content of some phenolic compounds to an increase in the relative abundance of others. Generally, the main changes involved the cleavage and assimilation of the sugar residue of some flavonol glycosides, resulting in the increased abundance of the aglycons quercetin and kaempferol. The fermentation of RODW with the tested common yeast strains had a similar overall effect on the phenolic composition. Contrary to the RODW-5 fermentation, no complete assimilation of particular phenolic compounds was observed for the tested common yeast strains, suggesting the better adaptation of the indigenous *Cyberlindnera* yeast to the fermentation of the phenolic glycosides present in RODW.

The biotransformation of flavonol glycosides was specific to the sugar residues involved. No new phenolic compounds were detected after the fermentation of RODW by the tested indigenous RODW isolates or common yeast strains. In a previous study, we proposed the recovery and valorization of RODW phenolics through their adsorption on a microporous resin [2]. The results from the present study suggest that spontaneous RODW fermentation by indigenous *Cyberlindnera* sp. yeasts, relating to a significant decrease in RODW sugars and phenolics, has to be taken into consideration when designing a large-scale RODW phenolic extraction. At the same time, the specific changes in the RODW phenolic composition following the fermentation could be used for the directional modification of the RODW composition and could be included as a preliminary step to increase the relative abundance and yield of specific phenolic compounds. Although the tested indigenous *Cyberlindnera* sp. isolates demonstrated superior fermentation rates of the RODW, the applicability of such fermentation to the remediation of RODW and phenolic removal prior to its release into the environment remains to be evaluated.

## 5. Conclusions

The phenolic-rich wastewater RODW generated after industrial rose oil distillation undergoes spontaneous fermentation by indigenous yeasts from the genus *Cyberlindnera*. The indigenous *Cyberlindnera* yeast isolates show superior (two to seven times higher) RODW fermentation capacity in comparison to all the tested common yeast strains. The fermentation of the RODW by indigenous *Cyberlindnera* yeasts results in a decrease in the phenolic content to around 60% of the initial concentration in the RODW and specific changes in the phenolic composition involving the biotransformation of particular phenolic compounds, mainly relating to the depletion and fermentation of the sugar moiety of the phenolic glycosides and an increase in the abundance of the corresponding aglycons. The results indicate that the studied indigenous *Cyberlindnera* yeast isolates could be further tested for the development of processes aimed at decreasing the content of phenolics in the RODW prior to its release into the environment or applied to the directional biotransformation of RODW and other plant phenolics before their extraction and valorization.

## Figures and Tables

**Table 1:** Relative amounts of 15 identified compounds in RODW phenolic extracts obtained after RODW fermentation with RODW-5 isolate and common industrial yeast strains. The relative amounts are presented as percentages of the amount of each compound in unfermented RODW analyzed in parallel. Each value is an average ± SD of the results from three independent RODW cultivations followed by phenolic extraction and HPLC analysis.

№	Compound	RODW-5	*Pichia* *angusta* ATCC 34438	*Pichia bispora* ATCC 10627	*Pichia guilliermondi* ATCC 9058	*Geotrichum fermentans* ATCC 10675	*Pichia anomala* ATCC 16763	*Zygoascus hellenicus* ATCC 15542
1	Elagic acid	29 ± 3 ab	102 ± 26 a	81 ± 8 ab	93 ± 8 a	39 ± 5 a	92 ± 13 abc	81 ± 36 abc
2	Hyperoside	237 ± 31 d	131 ± 17 ab	149 ± 11 ab	130 ± 10 a	150 ± 33 bcd	104 ± 12 abc	99 ± 27 abcd
3	Isoquercitrin	0 ± 0 a	127 ± 16 ab	5 ± 1 a	117 ± 9 a	86 ± 25 ab	95 ± 10 abc	99 ± 32 abcd
4	Kaempferol-3-O-rutinoside	225 ± 29 d	133 ± 18 ab	155 ± 7 abc	135 ± 10 ab	171 ± 19 cd	108 ± 12 abc	122 ± 21 bcd
5	Kaempferol 3-O-galactoside	269 ± 35 d	145 ± 26 ab	171 ± 8 abc	140 ± 9 ab	177 ± 19 cd	106 ± 12 abc	130 ± 23 cd
6	Quercetin-O-methyl disaccharide	211 ± 32 cd	115 ± 8 ab	79 ± 4 ab	128 ± 9 a	121 ± 8 abc	132 ± 12 c	30 ± 5 a
7	Astragalin (kaempferol 3-O-glucoside)	17 ± 3 a	130 ± 16 ab	29 ± 9 ab	109 ± 8 a	136 ± 13 bc	101 ± 10 abc	119 ± 19 bcd
8	Quercitrin	264 ± 36 d	128 ± 17 ab	184 ± 47 bc	146 ± 11 ab	175 ± 20 cd	98 ± 12 abc	185 ± 33 d
9	Kaempferol-3-O-xyloside	213 ± 29 cd	125 ± 13 ab	67 ± 4 ab	134 ± 10 ab	131 ± 10 bc	137 ± 14 c	40 ± 6 ab
10	Multiflorin B	119 ± 13 bc	135 ± 16 ab	157 ± 15 abc	127 ± 9 a	173 ± 20 cd	103 ± 11 abc	122 ± 26 bcd
11	Kaempferol-3-O-arabinoside	545 ± 70 e	128 ± 15 ab	316 ± 24 c	139 ± 10 ab	231 ± 28 d	111 ± 11 abc	275 ± 54 e
12	Kaempferol-3-O-rhamnoside	0 ± 0 a	125 ± 14 ab	149 ± 15 ab	124 ± 10 a	166 ± 20 bcd	61 ± 6 ab	112 ± 16 abcd
13	Multiflorin A	0 ± 0 a	111 ± 16 ab	127 ± 9 ab	113 ± 9 a	141 ± 15 bc	48 ± 6 a	137 ± 23 cd
14	Quercetin	174 ± 27 cd	144 ± 17 ab	626 ± 72 d	188 ± 21 b	354 ± 47 e	124 ± 32 abc	116 ± 10 abcd
15	Kaempferol	470 ± 62 e	155 ± 20 b	1182 ± 192 e	406 ± 58 c	472 ± 66 f	136 ± 70 c	135 ± 55 cd

Mean values sharing the same letter in each column do not differ significantly at *p* < 0.05.

## Data Availability

All obtained sequences were submitted to GenBank with the following accession numbers: RODW sequences, ITS (KU216396-KU216406, KU216435, KU216438), 18S-rDNA (KU216431, KU216436), 26S-rDNA (KU216432, KU216433), TEF1a (KU216434, KU216437); DLRFR sequences, ITS (KU216412-KU216429); RFE sequences, ITS (KU216407-KU216411).

## Supplementary Materials

The following supporting information can be downloaded at: https://www.mdpi.com/article/10.3390/microorganisms11010201/s1. Figure S1: HPLC UV chromatograms of resin extract from RODW obtained after fermentation for a period of 136 h by (a) indigenous yeast isolate RODW-5, (b) common yeast strain *Geotrichum fermentans* and (c) control RODW without fermentation. Peak designation corresponds to the numbering in Table 1. Figure S2: Hierarchical clustering of the RODW phenolic compounds according to their content changes following fermentation by the RODW-5 isolate. The dendrogram was constructed using the nearest neighbor algorithm and based on data for the relative changes of phenolic compounds in the extract of fermented RODW compared to a control extract of unfermented RODW, presented in Table 1. Figure S3: Hierarchical clustering of the RODW-5 isolate and the studied common yeast strains according to the changes in the content of phenolic compounds following RODW fermentation. The dendrogram was constructed using the nearest neighbor algorithm and based on data for the relative changes in phenolic compounds in the extract of fermented RODW compared to a control extract of unfermented RODW, presented in Table 1.
