# Supplementary material for: Indigenous Yeasts from Rose Oil Distillation Wastewater and Their Capacity for Biotransformation of Phenolics
Source: Microorganisms. 2023 Jan 12;11(1):201. doi: 10.3390/microorganisms11010201 (PMC9865748; doi:10.3390/microorganisms11010201)
Supplement: Supplementary file 1 [file microorganisms-11-00201-s001.zip › Supplementary Materials Figure S2.pdf]

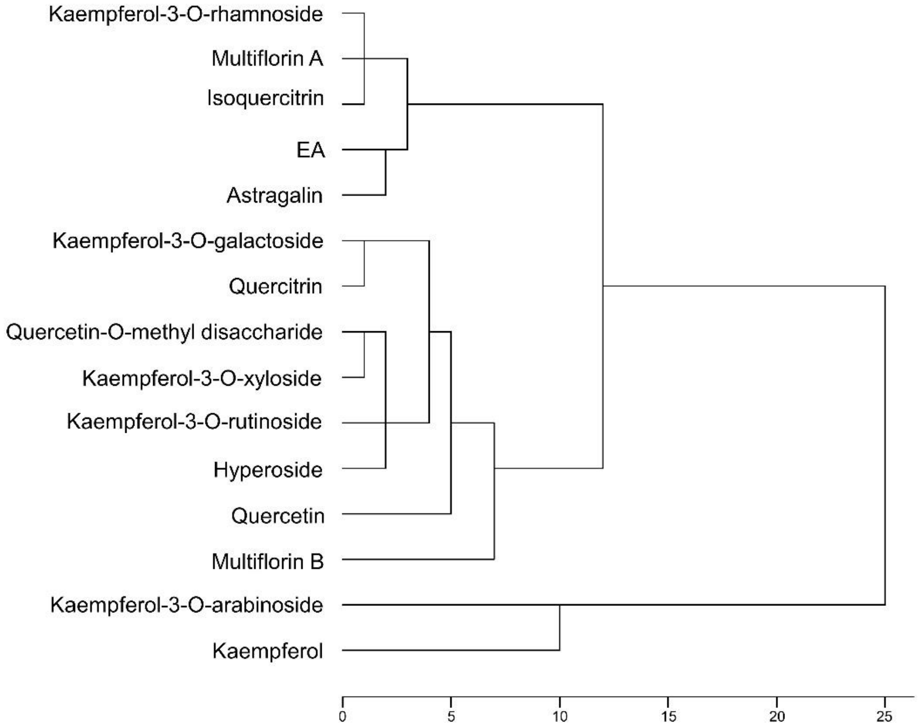

Figure S2. Hierarchical clustering of the RODW phenolic compounds according to their content changes following fermentation by the RODW-5 isolate. The dendrogram was constructed using the nearest neighbor algorithm and based on data for the relative changes of phenolic compounds in the extract of fermented RODW compared to a control extract of unfermented RODW, presented in Table 1.
