# Supplementary material for: Indigenous Yeasts from Rose Oil Distillation Wastewater and Their Capacity for Biotransformation of Phenolics
Source: Microorganisms. 2023 Jan 12;11(1):201. doi: 10.3390/microorganisms11010201 (PMC9865748; doi:10.3390/microorganisms11010201)
Supplement: Supplementary file 1 [file microorganisms-11-00201-s001.zip › Supplementary Materials Figure S3.pdf]

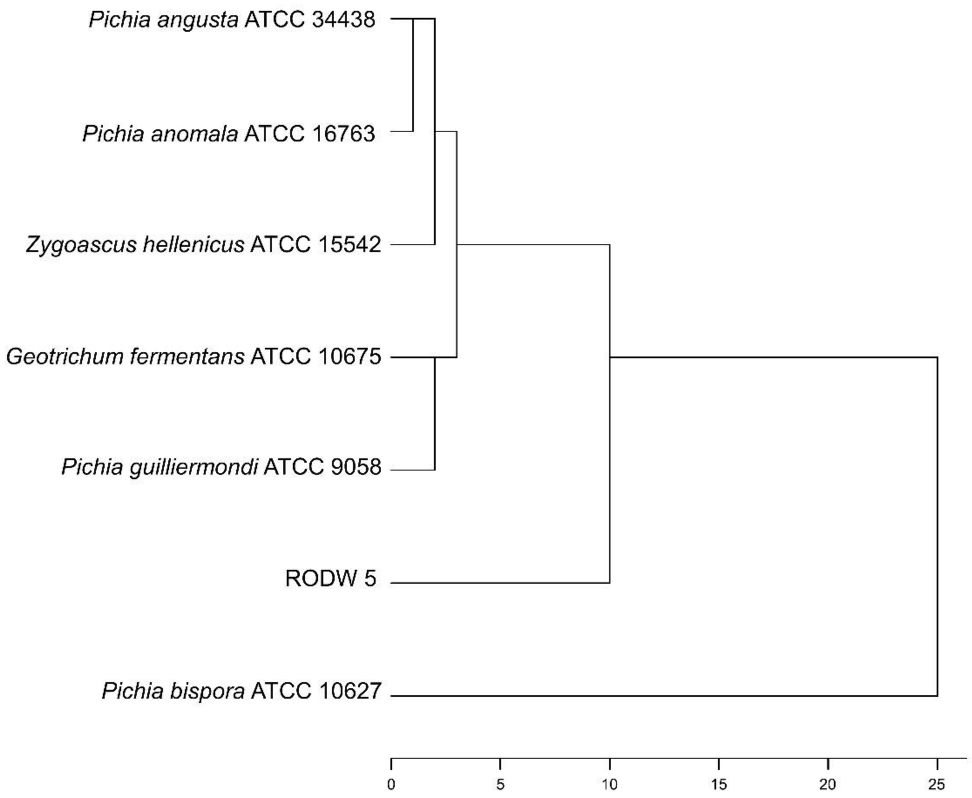

Figure S3. Hierarchical clustering of the RODW-5 isolate and the studied common yeast strains according to the changes in the content of phenolic compounds following RODW fermentation. The dendrogram was constructed using the nearest neighbor algorithm and based on data for the relative changes in phenolic compounds in the extract of fermented RODW compared to a control extract of unfermented RODW, presented in Table 1.
