# Supplementary material for: Indigenous Yeasts from Rose Oil Distillation Wastewater and Their Capacity for Biotransformation of Phenolics
Source: Microorganisms. 2023 Jan 12;11(1):201. doi: 10.3390/microorganisms11010201 (PMC9865748; doi:10.3390/microorganisms11010201)
Supplement: Supplementary file 1 [file microorganisms-11-00201-s001.zip › Supplementary Materials_Figure S1.pdf]

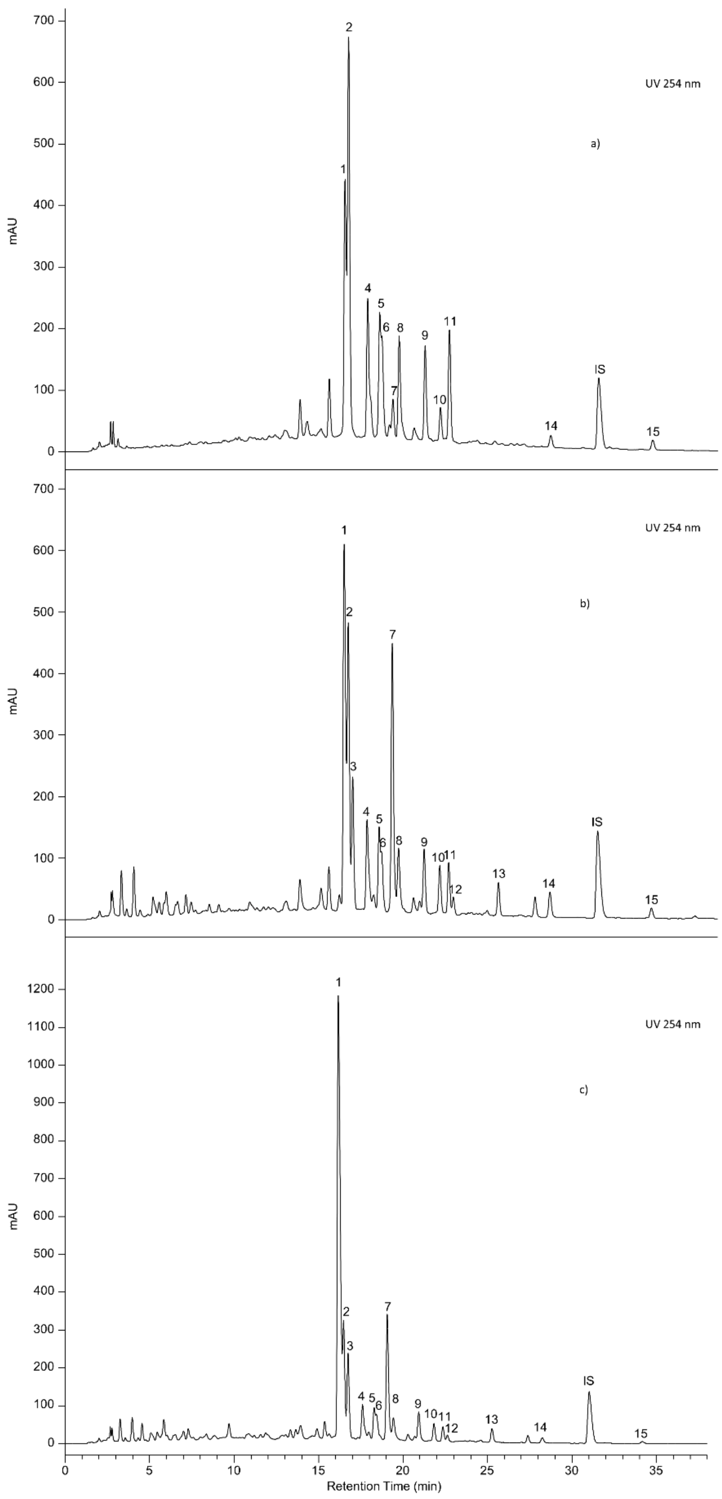

Figure S1. HPLC UV chromatograms of resin extract from RODW obtained after fermentation for a period of 136 hours by a) indigenous yeast isolate RODW-5, b) common yeast strain *Geotrichum fermentans* and c) control RODW without fermentation. Peak designation corresponds to the numbering in Table 1.
